# A233 OUTCOMES OF CLOSTRIDIOIDES DIFFICILE INFECTION IN SURGICAL PATIENTS: RESULTS FROM NATIONAL INPATIENT SAMPLE 2016-2019

**DOI:** 10.1093/jcag/gwac036.233

**Published:** 2023-03-07

**Authors:** R Tariq, M W Tahir, S Khanna

**Affiliations:** 1 Mayo Clinic; 2 Rochester General Hospital, Rochester, United States

## Abstract

**Background:**

Clostridioides difficile infection (CDI) is the most common nosocomial infection and is associated with significant morbidity and mortality.

**Purpose:**

We aimed to identify the burden of CDI in patients undergoing common gastrointestinal (GI) surgical procedures in a national inpatient cohort, as GI surgeries have been thought to be a risk factor for CDI.

**Method:**

We used the National Inpatient Sample (NIS) database from Unites States for the years 2016-2019 for this study. We included adult patients (age ≥ 18), who underwent common GI surgeries (identified using ICD-10 procedure codes), and among them identified patients with diagnosis of CDI. Outcomes assessed included risk of CDI among different surgeries, inpatient mortality, length of stay (LOS) and cost of hospitalization using regression analyses

**Result(s):**

From 2016 to 2019, an estimated total of 4,438,778 patients were hospitalized and underwent any of the studied GI surgeries. CDI was reported in 32,180 admissions (0.72%). Median age for CDI was higher than non-CDI patients (66 vs 56, p<0.001). Incidence of CDI was 2.5 times higher in patients admitted emergently compared to elective admissions (1.00% vs 0.40%, p<0.0001). Among all surgeries, the incidence of CDI was the highest for small bowel resection at 2.1% followed by partial esophagectomy at 1.6% and partial colectomy at 1.4%. Logistic regression analysis showed the patients undergoing esophagectomy had the highest risk with adjusted Odds Ratio (aOR) of 2.48, (95% CI 2.01 – 3.07, p<0.0001), followed by pancreatectomy with aOR 2.03 (95% CI 1.91 – 2.16, p<0.0001). Overall, surgical patients with CDI had a significantly higher in-patient mortality compared to non-CDI patients (8.2% vs 1.4%, p<0.0001). Logistic regression analysis showed an increased risk of inpatient mortality with CDI, with aOR 1.36, 95% CI 1.30 – 1.42, p<0.0001. Median LOS for surgical patients with CDI was higher than non-CDI patients (14 days vs 3 days, p<0.0001). The linear regression analysis for length of stay showed that among patients undergoing surgical procedures, CDI was associated with an increased LOS with beta of 8.39 days ± SE 0.04 (95% CI 8.31 – 8.46, p<0.0001). The mean cost of hospitalization for surgical patients with CDI was higher than non-CDI patients ($90,590 vs $31,702, p<0.0001) after adjusting for inflation over the four-year period. The linear regression analysis showed that CDI was associated with an increased cost of hospitalization with a beta of $25,343 ± SE 166 (95% CI 25,017 – 25,670, p<0.0001).

**Conclusion(s):**

CDI among GI surgeries leads to increase in inpatient mortality, length of stay and cost of hospitalization. Although the rate of CDI is showing a downwards trend, its impact on these outcome measures makes it an important complication to prevent and promptly treat in these surgical patients.

**Please acknowledge all funding agencies by checking the applicable boxes below:**

None

**Disclosure of Interest:**

None Declared

